# Responsiveness of Objective vs. Clinical Balance Domain Outcomes for Exercise Intervention in Parkinson's Disease

**DOI:** 10.3389/fneur.2020.00940

**Published:** 2020-09-25

**Authors:** Naoya Hasegawa, Vrutangkumar V. Shah, Graham Harker, Patricia Carlson-Kuhta, John G. Nutt, Jodi A. Lapidus, Se Hee Jung, Nancy Barlow, Laurie A. King, Fay B. Horak, Martina Mancini

**Affiliations:** ^1^Department of Neurology, Oregon Health and Science University, Portland, OR, United States; ^2^Department of Rehabilitation Science, Hokkaido University, Sapporo, Japan; ^3^Department of Rehabilitation Medicine, Seoul National University Boramae Medical Center, Seoul, South Korea

**Keywords:** Parkinson's disease, exercise, gait, anticipatory postural adjustments, automatic postural responses, objective measures, clinical measures, wearable technology

## Abstract

**Background:** Balance deficits in people with Parkinson's disease (PD) are often not helped by pharmacological or surgical treatment. Although balance exercise intervention has been shown to improve clinical measures of balance, the efficacy of exercise on different, objective balance domains is still unknown.

**Objective:** To compare the sensitivity to change in objective and clinical measures of several different domains of balance and gait following an Agility Boot Camp with Cognitive Challenges (ABC-C) intervention.

**Methods:** In this cross-over, randomized design, 86 individuals with PD participated in 6-week (3×/week) ABC-C exercise classes and 6-week education classes, consisting of 3–6 individuals. Blinded examiners tested people in their practical off state. Objective outcome measures from wearable sensors quantified four domains of balance: sway in standing balance, anticipatory postural adjustments (APAs) during step initiation, postural responses to the push-and-release test, and a 2-min natural speed walk with and without a cognitive task. Clinical outcome measures included the Unified Parkinson's Disease Rating Scale (MDS-UPDRS) Part III, the Mini Balance Evaluation Systems Test (Mini-BESTest), the Activities of Balance Confidence (ABC), and the Parkinson's Disease Questionnaire (PDQ-39). The standardized response means (SRM) of the differences between before and after each intervention compared responsiveness of outcomes to intervention. A linear mixed model compared effects of exercise with the active control—education intervention.

**Results:** The most responsive outcome measures to exercise intervention with an SRM > 0.5 were objective measures of gait and APAs, specifically arm range of motion, gait speed during a dual-task walk, trunk coronal range of motion, foot strike angle, and first-step length at step initiation. The most responsive clinical outcome measure was the patient-reported PDQ-39 activities daily living subscore, but all clinical measures had SRMs <0.5.

**Conclusions:** The objective measures were more sensitive to change after exercise intervention compared to the clinical measures. Spatiotemporal parameters of gait, including gait speed with a dual task, and APAs were the most sensitive objective measures, and perceived functional independence was the most sensitive clinical measure to change after the ABC-C exercise intervention. Future exercise intervention to improve gait and balance in PD should include objective outcome measures.

## Introduction

Balance dysfunction is one of the characteristic features of Parkinson's disease (PD) and emerges early, with subtle changes present already at the time of diagnosis (1). Balance dysfunction in people with PD includes impairments in many domains of balance control: (1) postural sway during quiet stance (Sway), (2) automatic postural responses (APRs) to external perturbations, (3) anticipatory postural adjustments prior to gait initiation (APAs), and (4) dynamic balance during walking (Gait) (2).

Balance dysfunction in people with PD are notoriously difficult to treat and are not often helped by pharmacological or surgical treatment, while there is evidence that exercise can improve mobility problems in people with PD. Two recent review papers summarized the effects of exercise intervention in people with PD on balance outcomes (3, 4). Both reviews showed improvements in clinical balance and gait outcomes measures, such as gait speed, the Berg Balance Scale (BBS) (5), disease severity (as measured by the Part III of the Unified Parkinson's disease Rating Scale, UPDRS), and activities of daily living (ADL). However, both reviews showed that exercise outcome measures for PD were limited to a stopwatch measure of gait speed and the BBS as a clinical balance scale but did not investigate the effects of exercise on specific balance domains. Clinical measures of balance or disease severity, such as the BBS or UPDRS, may not be sensitive to change with exercise and do not reflect improvements across specific balance domains (6). Only one recent study investigated the effects of exercise for people with PD using the subscores of the Mini Balance Evaluation Systems Test (Mini-BESTest) (7), a clinical scale that includes four balance domains: anticipatory postural adjustments, automatic postural responses, postural sway in stance in different sensory conditions, and gait (8). The results showed that a muscle strengthening program improved three subscores of the Mini-BESTest, excluding the Gait subscore, in people with PD, but the changes in Mini-BESTest were not achieved at the minimal clinically important difference (MCID) (9).

Objective measures of balance have been shown to be more sensitive to subtle impairments than clinical balance measures in people with PD (10, 11). Recently, wearable sensor systems have been shown to be useful to obtain objective measures across different balance domains in clinical settings due to their portability and quick objective analysis capability (12, 13). Recently, we reported clinimetric properties for objective measures of the four domains of balance (Sway, APRs, APAs, and Gait) from six wearable sensors worn on the feet, wrists, sternum, and lumbar spine (13–16). For example, we have shown that levodopa improves speed of gait and APAs but worsens postural sway instance (17). However, it is still unclear which specific objective measures of balance and gait would be useful as outcome measures for balance exercise intervention in people with PD. Previous studies showed that objective gait measures, but not clinical measures of balance or PD (such as the Mini-BESTest and UPDRS), were improved by dance, treadmill, or multimodal training (18, 19). However, it is unclear whether objective measures across all domains of balance are more sensitive than clinical measures to exercise intervention.

Our group recently showed that an Agility Boot Camp training incorporating cognitive challenges (ABC-C) (20–23) resulted in specific improvements in the APAs domain, measured by the Mini-BESTest, and improvements in clinical measures, such as the Postural Instability and Gait Difficulty (PIGD) score in the MDS-UPDRS, Quality of Life [the Parkinson's Disease Questionnaire-39 (PDQ-39) activities daily living (ADL) subscore] (20), as well as dual-cost of gait speed in people with PD (20, 22). Although we reported changes after the ABC-C intervention only in the APAs domain of the Mini-BESTest, we did not previously evaluate the effects of the ABC-C intervention for any objective measures of balance domains.

Thus, in this exploratory analysis, we compared the effects of the ABC-C intervention on clinical vs. objective outcome measures of balance using the four domains of balance (Sway, APRs, APAs, and Gait) within the Mini-BESTest (13–16). To narrow down the total number of objective measures for the four domains, we used those objective measures that recently were found to better discriminate between people with PD and healthy controls (24).

The purposes of this exploratory analysis are (1) to investigate which specific balance domains improved with the ABC-C intervention by using objective measures and (2) to compare responsiveness to the ABC-C intervention of objective vs. clinical outcome measures. We hypothesized that (1) three of four main balance domains that were part of the intervention (not APRs as postural responses were not practiced) would improve and (2) objective outcome measures of balance would be more sensitive than clinical outcome measures for the ABC-C intervention. We also related the most sensitive objective mobility measures to perceived change in Mobility and ADL and calculated the MCID.

## Methods

### Participants

Details on the participants' characteristics are reported in a previous publication by Jung et al. (20). Briefly, 94 individuals with idiopathic PD were enrolled in this study. Inclusion criteria were the following: (a) age between 50 and 90 years old, (b) no major musculoskeletal or peripheral or central nervous system disorders (other than PD) that could significantly affect their balance and gait, (c) ability to stand and walk unassisted, (d) no recent changes in medication (6 weeks of stable medications), and (e) meet criteria for idiopathic PD according to the Brain Bank Criteria for PD (25). Exclusion criteria were any other neurological disorders or musculoskeletal impairments that interfere with gait or balance and the inability to follow procedures. All participants signed informed consent forms approved by the Oregon Health & Science University institutional review board (approval no. 4131) and the joint OHSU and Veterans Affairs Portland Health Care System (VAPORHCS) institutional review board (approval no. 8979). All work was conducted in accordance with the declaration of Helsinki (1964). This trial was registered on Clinical Trials.gov (NCT02231073 and NCT02236286).

### Procedure

A cross-over, randomized, controlled trial design of a 6-week ABC-C intervention for people with PD was conducted from 2014 to 2018 (21). Participants were randomized into one of two intervention groups, Exercise First or Education First, by a computerized block randomization. The researchers who performed and analyzed all baseline, midpoint, and final tests remained blinded to group assignment throughout the duration of the study. Individuals randomized to Exercise First participated in a 6-week ABC-C intervention and crossed over to receive the 6-week education intervention, and individuals in Education First participated in an education class and crossed over to receive ABC-C intervention. Both interventions were designed to have similar frequency and delivered by the same exercise trainers. More details are reported in Jung et al. (20).

The following clinical scales and questionnaires were used as outcome measures for this analysis: (1) Mini-BESTest, (2) MDS-UPDRS (26), (2) the Activities-Specific Balance Confidence scale (ABC-scale) (27), (3) the Montreal Cognitive Assessment (MoCA) (28), (4) the New Freezing of Gait Questionnaire (NFOGQ) (29), and (5) PDQ-39 (30).

**Figure 1:**
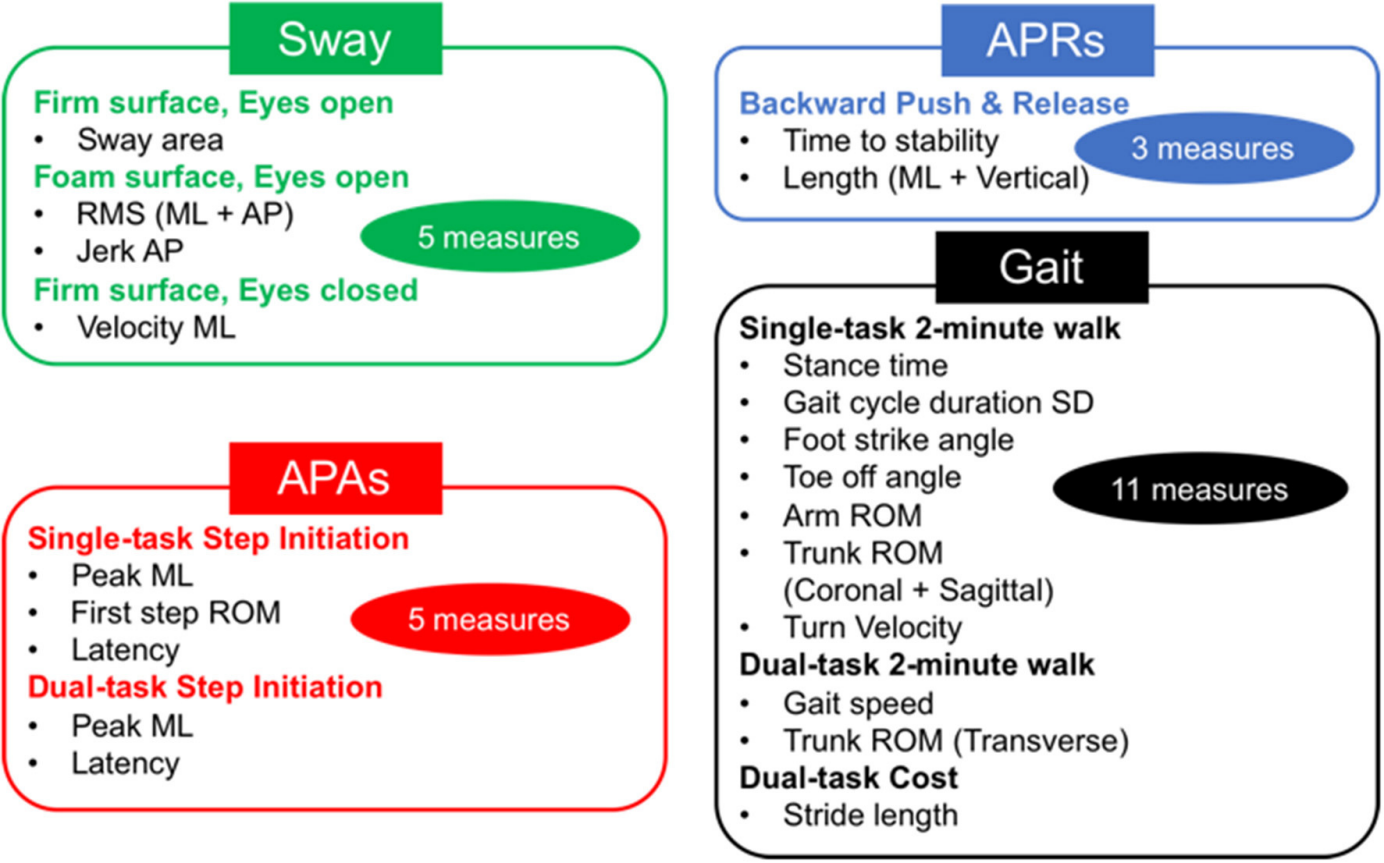
Total objective measures 24. Twenty-four sensitive objective measures have been selected to discriminate between people with Parkinson's disease (PD) and healthy elderly in four postural control domains (20). APAs, anticipatory postural adjustments; APRs, automatic postural responses; ML, mediolateral; AP, anteroposterior; ROM, range of motion; SD, standard deviation.

Objective measures of balance were obtained via six wearable sensors (Opals, APDM), each including triaxial accelerometers, triaxial gyroscopes, and magnetometers, placed on both feet, wrists, sternum, and the lumbar region, while performing a total of eight different motor tasks, summarized below and in Figure 1. Participants were tested in their practical Off state after at least 12 h of medication washout. The same battery of clinical and mobility measurements was carried out after 6 weeks of intervention before the participants crossed over into the second intervention and again at the end of the second intervention.

The protocol for both the ABC-C and Education interventions has been detailed in our previous studies (20–23). Briefly, the ABC-C intervention consisted of a 90-min group exercise session, 3 days per week for 6 weeks, led by a certified exercise trainer. The program included the following: (1) gait training, (2) functional skill training (31), (3) agility course, (4) lunges, (5) boxing, and (6) adapted tai chi (32). Each exercise was engaged for 10–20 min with rest periods in between the exercises (21, 23). Each exercise was systematically progressed from beginning to intermediate to advanced levels by challenging (a) divided attention with secondary cognitive tasks, (b) response inhibition, (c) limiting external sensory cues, (d) increasing the length, complexity, and novelty of whole-body movement sequences, and (e) increasing repetitions, speed, amplitude, resistance, or balance requirements.

In the Education intervention, participants were taught how to live better with their chronic conditions. Classes consisted of a group of participants (up to six) meeting with the same trainer for a 90-min session, once a week for 6 weeks. In order to match the dose of the Education intervention with the ABC-C intervention, participants were provided relaxation tapes to be used at home five times per week for 30 min for an overall education dose of 240 min, similar to the exercise dose. Compliance was recorded for both the ABC-C and Education intervention at each session. The trainer coded the progression of exercise difficulty at the end of each week to determine the level of exercise progression for each participant. Additionally, the level of self-reported exertion (0–10) was recorded to determine the level of challenge of the program and to determine if people were progressively challenged during the exercise over time.

### Outcome Measures

The full protocol of mobility tasks has been detailed in our previous study (24). The eight motor tasks included Sway, APRs, APAs, and Gait tasks (see Figure 1). The Sway task consisted of standing still for 30 s on a firm surface with eyes open or closed (EOFirm and ECFirm), and on a foam surface with eyes open (EOFoam). The APRs task consisted of the push and release test in the backward direction (14). An Instrumented Stand and Walk test (15) and a 2-min walk test were used to extract measures of APAs and Gait, respectively. In addition, both APAs and Gait task were performed with and without a concurrent cognitive task (single and dual task) (24). The dual-task condition consisted of serial subtraction by threes from a three-digit number, during both quiet stance and during the gait initiation (APA task) and in reciting every other letter of the alphabet while walking for the Gait task. As objective outcome measures, we used 24 objective measures that were found to be most sensitive in discriminating between people with PD and healthy controls as determined from our previous study (24) (see details in Figure 1). When a Dual task was added, the dual-task cost (DC) was calculated as DC (%) = 100 × (dual-task measure – single-task measure)/single-task measure.

The clinical Mini-BESTest and its four subscores (APAs, APRs, Sway, and Gait) were assessed as a clinical measure of dynamic balance. The total of MDS-UPDRS and the subtotal of Parts II and III were used as measures of disease severity, and the PIGD subscore (sum of items 3.9, 3.10, 3.12, and 3.13 of the MDS-UPDRS) was calculated to assess disease severity focusing on balance. The MoCA score was used as a measure of general cognition. The ABC scale was used to assess balance confidence and balance self-perception. The total PDQ-39 and the Mobility/ADL subscores provided patient-reported quality of life. Lastly, the perceived change in Mobility and ADL after Exercise and Education were determined at the second and third observation according to the following scale: (3) excellent improvement, (2) moderate improvement, (1) mild improvement, (0) no change, (−1) mild worsening, (−2) moderate worsening, and (−3) terrible worsening. For the perceived change in Mobility and ADL, participants were asked the following: “Did you notice a change in the past 6 weeks in your balance and gait?” and “Did you notice a change in the ability to carry out your daily activities in the past 6 weeks?” To determine the MCID of objective measures, the scores after the ABC-C intervention were used for the statistical analysis.

### Statistical Analysis

The distribution for each demographic and clinical measure of the two groups (Exercise First/Education First) was examined by the Shapiro–Wilk test at baseline. For data that were nonnormally distributed, the Mann–Whitney *U*-test was used to determine a difference between groups at baseline. Otherwise, independent samples *t*-test and chi-squared tests were used to examine possible group differences at baseline.

To investigate whether outcome measures differed between each intervention, a linear mixed model was fit for each objective measure. Since we had three observations for each participant (baseline, midpoint, and final), we calculated the changes due to the ABC-C intervention as midpoint–baseline for the Exercise first group and final–midpoint for the Education first group. Similarly, the changes due to the Education intervention were calculated as final–midpoint for the Exercise first group and midpoint–baseline for the Education first group. The linear mixed-model design included an indicator of intervention effects (Education vs. Exercise), order effects (Exercise or Education first), and period effects (sequence, Education–Exercise or Exercise–Education, differences) to determine whether the “difference in change” differed between Exercise and Education. The intervention term reflected whether the effects of Exercise differed from the effects of Education. A random effects term was included for participants. In addition, the effect of Exercise and Education were calculated as standardized response mean (SRM) for each clinical and objective measure. The SRM was calculated as the mean change between before and after each intervention period divided by the standard deviation (SD) of the change (33). An SRM value of 0.20 represents a small, 0.50 a moderate, and 0.80 a large effect of the intervention (33).

Last, the MCID of the objective measures with a significant difference between both interventions was determined by using two different types of anchor-based approaches based on the perceived change in Mobility or ADL (9, 34). One of the methods to define the MCID was that the delta of objective measures associated with the perceived change in Mobility or ADL 0 (no change) were compared with the delta of objective measures associated with the perceived change in Mobility or ADL 1 (mild improvement) (34). The other anchor-based method used receiver operating characteristic (ROC) curve technique to find the most suitable MCID values following the method described by Hauser et al. (35). Assuming that false-positive and false-negative identifications are equally unwanted, we determined the cutoff value with the most optimal balance between sensitivity and specificity. The optimal cutoff point to distinguish the delta of objective measures between subjects rated as unchanged (value of 0) from subjects rated as mild improvement (value of 1) was estimated as the point on the ROC curve closest to the point of (0,1). It was calculated as the minimum value of the following formula:

$$\begin{array}{l} The\;value=\;\sqrt{{\left(1-Sensitivity\right)}^{2}+{\left(1-Specificity\right)}^{2}} \end{array}$$

For the most optimal cutoff values, the positive (LR+) and negative (LR–) likelihood ratios were also determined using the following formulas:

$$\begin{array}{l} LR+=\frac{True\;positive\;rate}{False\;positive\;rate}=\;\frac{Sensitivity}{\left(1-Specificity\right)}\\ LR-=\frac{False\;negative\;rate}{True\;negative\;rate}=\;\frac{\left(1-Sensitivity\right)}{Specificity} \end{array}$$

Furthermore, the area under the curve was calculated to compare the accuracy of the prediction for the perceived change. An area under the curve (AUC) value of 0.56 represents a small, 0.64 a moderate, and 0.71 a high accuracy of the prediction for perceived change (36). Prior to determining the MCID, the association between delta of the objective measures, and the perceived change in Mobility or ADL was calculated using Spearman's rho correlation coefficient. The MCID was detected for the delta of mobility measures that correlated with the perceived change in Mobility or ADL (*r* > 0.3) (34).

The statistical analysis for the demographic data and clinical measures at baseline and association between delta and the perceived change were processed using SPSS Statistics version 25.0 (IBM, Armonk, NY, USA), and a linear mixed model was calculated using MATLAB R2018b (The Mathworks Inc., Natick, MA, USA) with the Statistics and Machine Learning Toolbox. The statistical significance for this exploratory analysis was set to *p* < 0.01.

## Results

Ninety-four participants were randomly assigned into two groups [Exercise First: *n* = 46; Education First: *n* = 45; see cohort diagram in Jung et al. (20)]. Further analysis were performed on the 86 participants who had at least two data points (Exercise First: *n* = 44; Education First: *n* = 42). Age, height, weight, and gender were not different between the Exercise First and Education First groups at baseline (Table 1). In addition, there were no significant differences between the Exercise First and Education First group in disease severity (MDS-UPDRS, Hoehn and Yahr stage, and the ratio of freezers), clinical balance function (Mini-BESTest), perceived functional independence (PDQ-39), or general cognitive function (MoCA) before participating this study (details in Table 1).

**Table 1:** Demographic data.

	**All** ** (*****N*** **=** **86)**		**Exercise First** ** (*****N*** **=** **44)**		**Education First** ** (*****N*** **=** **42)**		
	**Mean**	***SD***	**Mean**	***SD***	**Mean**	***SD***	***p*-value**
Male/Female	58/28		30/14		28/14		0.881^a^
Age	68.8	7.6	67.7	6.7	70.0	8.2	0.152
Height (cm)	174.0	9.6	174.0	10.3	174.1	8.9	0.997^b^
Weight (kg)	79.4	15.3	81.5	15.6	77.2	14.7	0.195
Disease Duration (years)	6.5	5.0	6.2	4.4	6.7	5.5	0.921^b^
**MDS-UPDRS**							
Total	68.2	20.4	67.2	20.2	69.3	20.7	0.651
Part III	42.3	12.2	40.7	11.1	43.9	13.1	0.232
PIGD score	5.4	2.8	4.9	2.5	5.9	3.0	0.094^b^
Mini-BESTest	18.1	4.8	18.6	4.3	17.5	5.2	0.438^b^
ABC scale	80.4	16.0	80.3	17.7	80.4	14.0	0.635^b^
PDQ-39	16.5	11.6	16.7	11.5	16.3	11.8	0.788^b^
MoCA	25.6	3.5	26.5	2.9	24.6	3.9	0.016^b^
Hoehn and Yahr stage	1/69/8/8		1/38/4/1		0/31/4/7		0.104^a^
(I/II/III/IV)							
FoG/without FoG	42/44		23/21		19/23		0.514^a^

*Groups compared using independent sample t-test, Mann–Whitney U-test, or chi-squared test and significance level of 0.01*.

a *Chi-squared test*.

b *Mann–Whitney U-test*.

*PD, Parkinson's disease; MDS-UPDRS, Movement Disorder Society-Sponsored Revision of the Unified Parkinson's Disease Rating Scale; PIGD, postural instability and gait disability; Mini-BESTest, mini Balance Evaluation Systems Test; ABC scale, the Activities-Specific Balance Confidence scale; PDQ-39, Parkinson's Disease Questionnaire-39; MoCA, Montreal Cognitive Assessment; FoG, Freezing of Gait*.

**Figure 2:**
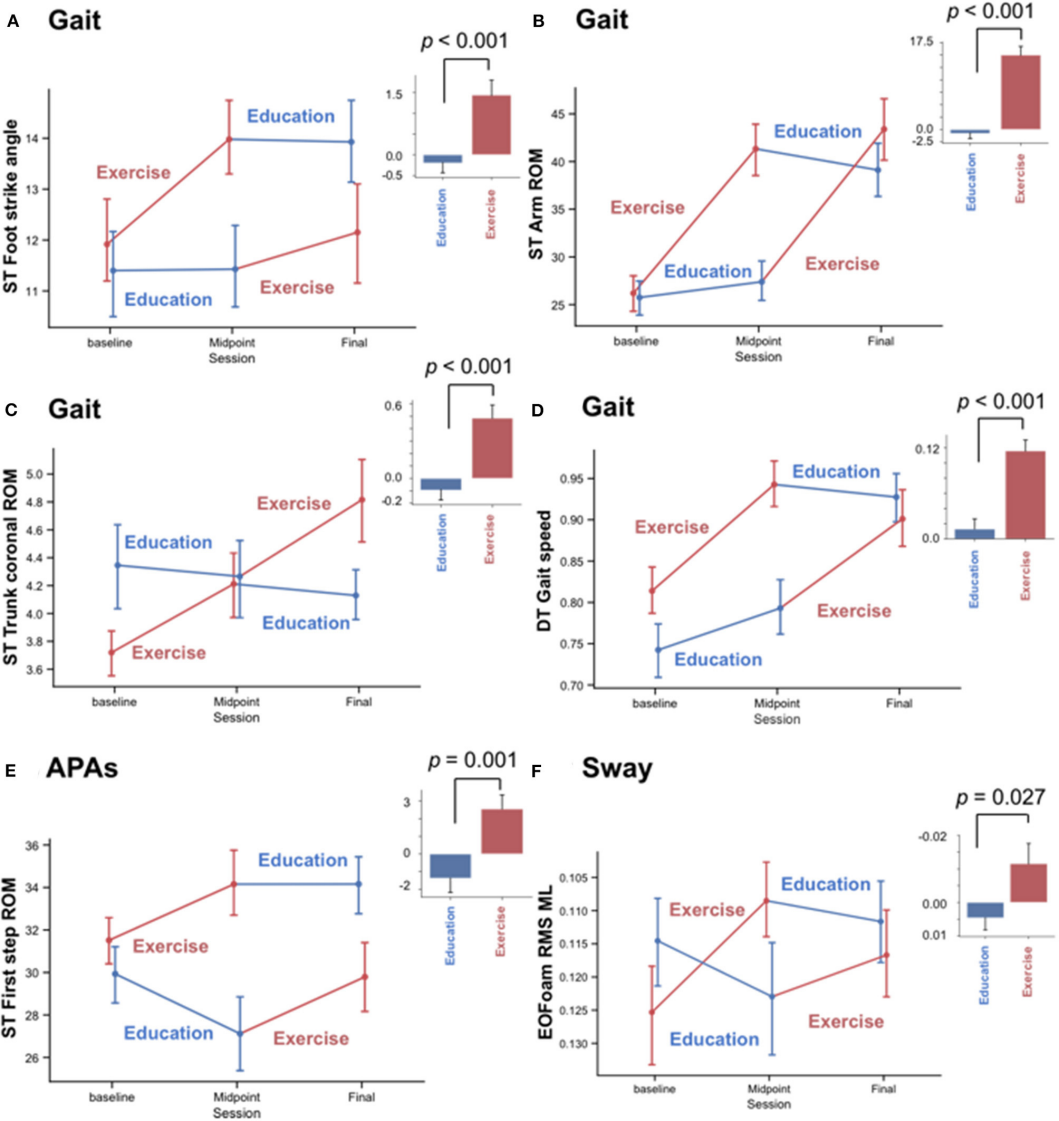
Significant effects of the Exercise but not Education intervention on objective measures of gait and anticipatory postural adjustments (APAs), but not sway. Mean and standard error of mean (SEM) plots of **(A–D)** four gait measures and **(E,F)** two balance measures. **(A)** Foot strike angle, **(B)** Arm range of motion (ROM), and **(C)** trunk coronal ROM, **(D)** gait speed during a dual-task walk (DT), **(E)** anticipatory postural adjustments (APAs) involving first-step ROM at step initiation during a single-task walk (ST), and **(F)** root mean square (RMS) of medio-lateral (ML) sway while standing on a foam surface with eyes open (EOFoam). Plots divide results into the randomized Exercise First and Education First groups with Exercise intervention in red and Education intervention in blue. Histograms summarize the change in each measure before vs. after the Education and Exercise intervention. Error bar shows SEM, and *p*-value was calculated by a linear mixed model.

The objective measures showing significant improvements after the ABC-C intervention compared to the Education intervention were in the domains of Gait and APAs (see Tables 2, 3 and Figure 2). Specifically, arm swing ROM, foot strike angle, and trunk coronal ROM during single-task walking significantly increased after the ABC-C intervention compared to the Education intervention (*p* < 0.001, Table 2). In addition, gait speed during dual-task walking was significantly faster after the ABC-C intervention compared to the Education intervention (*p* < 0.001). Lastly, both the peak ML and the first-step ROM during gait initiation were significantly larger after the ABC-C intervention compared to the Education intervention (*p* = 0.003 and *p* = 0.001). None of these measures showed a significant order or period effect (*p* > 0.01). However, two objective measures in the Gait domain, stance time, and toe-off angle showed a significant period effect (*p* < 0.01) in the absence of a significant intervention effect (Table 2). In contrast to Gait and APAs, measures of Sway and APRs did not change (*p* > 0.01, Table 2).

**Table 2:** Means and standard deviations (SDs) of each outcome measures at baseline and changes at 6-weeks for Education and ABC-C. Standardized Response Mean with confidence intervals is reported.

**Balance** ** domain**	**Objective measure**	**Baseline**		**Change after 6-week education**					**Change after 6-week ABC-C**				
		**Mean (SD)**		**Mean (SD)**		**SRM**	**Lower *CI***	**Upper *CI***	**Mean (SD)**		**SRM**	**Lower *CI***	**Upper *CI***
Gait	Arm ROM (degree)	26.19	12.09	−0.81	9.37	−0.09	−0.30	0.13	15.02	10.89	0.95	0.68	1.21
	DT Gait speed (m/s)	0.78	0.2	0.01	0.12	0.11	−0.11	0.33	0.12	0.1	0.94	0.67	1.21
	Trunk coronal ROM (degree)	4	1.61	−0.09	0.73	−0.13	−0.35	0.09	0.48	1.07	0.45	0.22	0.68
	Foot strike angle (degree)	11.68	5.46	−0.2	2.29	−0.09	−0.30	0.13	1.44	3.2	0.45	0.22	0.68
	Toe off angle (degree)	30.04	4.66	0.12	1.89	0.07	−0.15	0.28	0.84	1.93	0.43	0.20	0.66
	DC Stride length (%)	−10.6	7.53	0.84	8.6	0.10	−0.12	0.32	2.83	7.14	0.4	0.16	0.63
	Trunk sagittal ROM (degree)	3.78	0.86	0.11	0.68	0.17	−0.05	0.39	0.31	0.85	0.37	0.14	0.59
	Stance time (%)	61.37	1.92	−0.17	0.89	−0.2	−0.41	0.02	−0.47	1.31	−0.36	−0.58	−0.13
	Gait cycle duration SD (s)	0.04	0.02	0	0.01	−0.07	−0.28	0.15	0	0.01	−0.14	−0.36	0.08
	DT Trunk transverse ROM (degree)	6.96	2	0.03	1.73	0.02	−0.20	0.24	0.15	1.73	0.09	−0.14	0.31
	Turn velocity (degree/s)	134.85	35.28	2.26	17.31	0.13	−0.09	0.35	0.75	23.84	0.03	−0.19	0.25
Sway	EOFoam RMS ML (m/s^2^)	0.121	0.046	0.005	0.036	0.13	−0.11	0.36	−0.012	0.046	−0.25	−0.50	−0.01
	EOFoam Jerk AP (m/s^5^)	8.08	10.26	1.23	13.97	0.09	−0.15	0.33	−3.45	15.9	−0.22	−0.46	0.03
	ECFirm Velocity ML (m/s)	0.125	0.078	0.003	0.092	0.03	−0.19	0.26	−0.019	0.101	−0.19	−0.42	0.04
	EOFoam RMS AP (m/s^2^)	0.132	0.047	0.002	0.064	0.03	−0.21	0.26	−0.012	0.073	−0.16	−0.40	0.08
	EOFirm Sway area (m/s^2^)	0.095	0.062	0	0.057	0.01	−0.22	0.23	0.007	0.059	0.12	−0.11	0.36
APAs	First step ROM (degree)	30.25	8.63	−1.37	8.09	−0.17	−0.39	0.05	2.55	7.48	0.34	0.11	0.57
	peak ML (m/s^2^)	0.032	0.013	−0.003	0.016	−0.21	−0.43	0.02	0.005	0.018	0.28	0.05	0.51
	Latency (s)	0.72	0.28	0.03	0.226	0.13	−0.09	0.36	−0.034	0.25	−0.14	−0.36	0.09
	DT Latency (s)	0.74	0.21	0.052	0.342	0.15	−0.08	0.38	0.031	0.221	0.14	−0.10	0.38
	DT peak ML (m/s^2^)	0.031	0.016	−0.001	0.014	−0.05	−0.28	0.18	0	0.016	0	−0.23	0.24
APRs	Length ML (m)	0.153	0.11	0.024	0.128	0.19	−0.05	0.43	−0.018	0.122	−0.14	−0.39	0.11
	Time to stability (s)	1.3	0.63	−0.079	0.662	−0.12	−0.34	0.14	−0.074	0.642	−0.12	−0.37	0.13
	Length vertical (m)	0.044	0.026	0.002	0.022	0.1	−0.16	0.32	−0.002	0.025	−0.09	−0.34	0.16

*Lower and upper 95% confidence intervals (CI) for standardized response mean (SRM) are also presented. Objective measures are arranged in descending order of SRM for Exercise intervention. ROM, Range of Motion; DT, Dual-task; DC, Dual-task Cost; EOFirm, Firm surface with Eyes Open; EOFoam, Foam surface with Eyes Open; ECFirm, Firm surface with Eyes Closed; RMS, Root Means Square; ML, Medio-Lateral; APAs, Anticipatory Postural Adjustments; APRs, Automatic Postural Responses*.

**Table 3:** Results from linear mixed models for the change of each objective measures after intervention.

**Balance domain**	**Measure**	**Fixed factor**	**Beta**	***t*-value**	**Lower CI**	**Upper CI**	***p*-value**
Gait	**Arm ROM (degree)**	**Intervention**	**−15.883**	**−7.88**	**−19.865**	**−11.902**	**<0.001**
		Order	2.958	1.468	−1.023	6.938	0.144
		Period	−2.057	−1.021	−6.037	1.922	0.309
	**DT Gait speed (m/s)**	**Intervention**	**−0.102**	**−5.412**	**−0.139**	**−0.065**	**<0.001**
		Order	0.016	0.834	−0.021	0.053	0.406
		Period	−0.043	−2.301	−0.08	−0.006	0.023
	**Trunk coronal ROM (degree)**	**Intervention**	**−0.58**	**−4.052**	**−0.862**	**−0.297**	**<0.001**
		Order	0.014	0.095	−0.269	0.296	0.925
		Period	−0.005	−0.032	−0.287	0.278	0.974
	**Foot strike angle (degree)**	**Intervention**	**−1.606**	**−3.75**	**−2.452**	**−0.76**	**<0.001**
		Order	−0.445	−1.04	−1.291	0.4	0.3
		Period	−0.862	−2.012	−1.707	−0.016	0.046
	Toe off angle (degree)	Intervention	−0.706	−2.414	−1.283	−0.128	0.017
		Order	0.176	0.602	−0.401	0.753	0.548
		Period	−0.79	−2.705	−1.368	−0.213	0.008
	DC Stride length (%)	Intervention	−2.021	−1.606	−4.507	0.465	0.11
		Order	1.293	1.028	−1.192	3.777	0.306
		Period	−0.127	−0.101	−2.612	2.358	0.92
	Trunk sagittal ROM (degree)	Intervention	−0.205	−1.714	−0.441	0.031	0.088
		Order	0.209	1.751	−0.027	0.445	0.082
		Period	−0.047	−0.393	−0.283	0.189	0.695
	Stance time (%)	Intervention	0.287	1.684	−0.05	0.623	0.094
		Order	−0.139	−0.815	−0.475	0.198	0.417
		Period	0.469	2.751	0.132	0.805	0.007
	Gait cycle duration SD (s)	Intervention	0.001	0.389	−0.003	0.005	0.698
		Order	−0.001	−0.513	−0.005	0.003	0.609
		Period	0.005	2.45	0.001	0.01	0.015
	DT Trunk transverse ROM (degree)	Intervention	−0.113	−0.41	−0.657	0.431	0.682
		Order	−0.16	−0.581	−0.704	0.384	0.562
		Period	0.031	0.112	−0.513	0.575	0.911
	Turn velocity (degree/s)	Intervention	1.754	0.544	−4.619	8.127	0.587
		Order	−0.575	−0.178	−6.945	5.795	0.859
		Period	−7.29	−2.261	−13.66	−0.92	0.025
Sway	EOFoam RMS ML (m/s^2^)	Intervention	0.016	2.233	0.002	0.029	0.027
		Order	0.005	0.719	−0.009	0.019	0.473
		Period	0.007	1.002	−0.007	0.021	0.318
	EOFoam Jerk AP (m^2^/s^5^)	Intervention	4.871	1.916	−0.159	9.901	0.058
		Order	−0.059	−0.023	−5.086	4.968	0.982
		Period	−3.604	−1.418	−8.632	1.424	0.159
	ECFirm Velocity ML (m/s)	Intervention	0.023	1.443	−0.008	0.054	0.151
		Order	0.01	0.652	−0.021	0.042	0.516
		Period	−0.015	−0.928	−0.046	0.017	0.355
	EOFoam RMS AP (m/s^2^)	Intervention	0.013	1.102	−0.01	0.036	0.272
		Order	0.013	1.122	−0.01	0.036	0.264
		Period	0.001	0.044	−0.023	0.024	0.965
	EOFirm Sway area (m/s^2^)	Intervention	−0.007	−0.713	−0.025	0.012	0.477
		Order	0.017	1.837	−0.001	0.036	0.068
		Period	0	0.004	−0.019	0.019	0.997
**APAs**	**First step ROM (degree)**	**Intervention**	**−3.94**	**−3.265**	**−6.323**	**−1.557**	**0.001**
		Order	−1.235	−1.024	−3.617	1.147	0.307
		Period	1.808	1.499	−0.574	4.19	0.136
	**Peak ML (m/s**^**2**^**)**	**Intervention**	**−0.008**	**−3.016**	**−0.013**	**−0.003**	**0.003**
		Order	−0.003	−1.305	−0.009	0.002	0.194
		Period	−0.007	−2.613	−0.012	−0.002	0.01
	Latency (s)	Intervention	0.066	1.719	−0.01	0.141	0.088
		Order	0.012	0.324	−0.063	0.088	0.747
		Period	−0.042	−1.087	−0.117	0.034	0.279
	DT Latency (s)	Intervention	0.023	0.47	−0.073	0.118	0.639
		Order	−0.003	−0.072	−0.099	0.092	0.943
		Period	−0.031	−0.641	−0.126	0.064	0.523
	DT peak ML (m/s^2^)	Intervention	−0.001	−0.234	−0.005	0.004	0.815
		Order	−0.004	−1.684	−0.009	0.001	0.094
		Period	−0.002	−0.619	−0.006	0.003	0.537
APRs	Length ML (m)	Intervention	0.043	1.969	0	0.086	0.051
		Order	0.007	0.304	−0.037	0.05	0.761
		Period	−0.02	−0.917	−0.063	0.023	0.361
	Time to stability (s)	Intervention	−0.007	−0.06	−0.233	0.219	0.952
		Order	−0.073	−0.638	−0.298	0.153	0.525
		Period	0.047	0.409	−0.179	0.272	0.683
	Length vertical (m)	Intervention	0.005	1.133	−0.003	0.012	0.259
		Order	0.003	0.686	−0.005	0.011	0.494
		Period	−0.005	−1.288	−0.013	0.003	0.2

*Values in bold indicate significant intervention effects at p < 0.01. Lower and upper 95% confidence intervals (CIs) for beta are also presented. Italic values indicate standardized response mean (SRM). Objective measures are arranged in descending order of SRM for Exercise intervention*.

*ROM, range of motion; DT, dual-task; DC, dual-task cost; EOFirm, firm surface with eyes open; EOFoam, foam surface with eyes open; ECFirm, firm surface with eyes closed; RMS, root mean square; ML, medio-lateral; APAs, anticipatory postural adjustments; APRs, automatic postural responses*.

**Figure 3:**
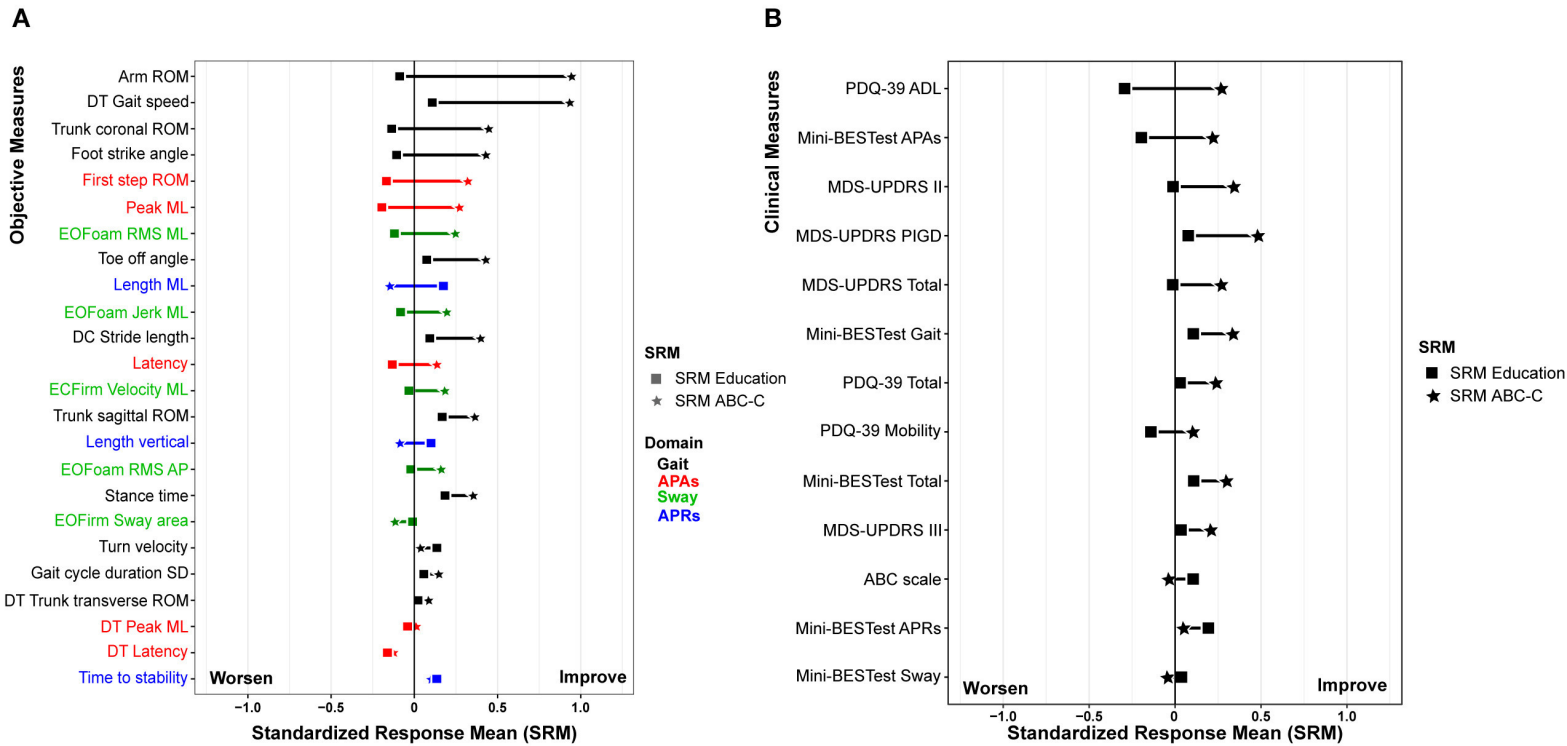
Effect size of the **(A)** objective measure and **(B)** clinical measure after the ABC-C Intervention (square) and Education intervention (star). All of the plots are displayed in descending order of the difference of standardized response means (SRM) between for the education and Agility Boot Camp with Cognitive Challenges (ABC-C) intervention.

Out of the Gait measures, arm swing ROM during single-task walking (SRM_ABC−C_ = 0.95, SRM_Education_ = −0.09), and gait speed during a dual-task walk (SRM_ABC−C_ = 0.94, SRM_Education_ = 0.11) showed the largest effect sizes after the ABC-C intervention but not after the Education intervention (Table 2 and Figure 3A). Foot strike angle (SRM_ABC−C_ = 0.45; SRM_Education_ = −0.09) and trunk coronal ROM (SRM_ABC−C_ = 0.45; SRM_Education_ = −0.13) during a single-task walk showed small effect size after the ABC-C intervention but not after the Education intervention.

The results of a linear mixed model for the clinical measures have been detailed in our previous paper Jung et al. (20). Figure 3B summarizes the effect size after the ABC-C and Education interventions on the clinical measures. All of the clinical measures showed small or no effect sizes after the ABC-C intervention compared to the objective measures.

Spearman's correlation coefficient showed that Arm ROM during a single-task walk and Gait speed during a dual-task walk were associated with the perceived change in ADL (rho = 0.36 and 0.46, respectively). In addition, Arm ROM during a single-task walk correlated with the perceived change in Mobility (rho = 0.37). Therefore, we calculated the MCID for these two objective measures. Based on the mean change approach, we found 23.0- and 21.2-degrees improvement as the MCID for Arm ROM during a single-task walk with SRM of 1.19 and 1.25 calculated by perceived change in Mobility and ADL, respectively. We also found a 0.14 m/s improvement as MCID Gait speed during a dual-task walk with SRM of 0.86 calculated by perceived change in ADL (Table 4). Based on the ROC approach, the best cut-off value discriminating no change from mild improvement in the perceived change in Mobility and ADL, respectively, was 17.7 and 17.2 with AUC of 0.64 and 0.67 for Arm ROM during a single-task walk. Furthermore, the best cutoff value to detect a perceived change in ADL was 0.13 m/s improvement for Gait speed during a dual-task walk with AUC of 0.67. Table 4 summarizes the MCID for Arm ROM during a single-task walk and Gait speed during a dual-task walk determined by two anchor-based approaches.

**Table 4:** Mean delta value of objective measures associated with the perceived change score.

			**Mean approach**					**ROC approach**					
**Objective measure**	**Perceived change of Mobility**		***N***	**Mean**	**Lower *CI***	**Upper *CI***	**SRM**	**Cutoff**	**Sensitivity**	**Specificity**	**LR +**	**LR –**	**AUC**
Arm ROM	1	Mild improvement	11	23.00	11.62	34.39	1.19	17.66	0.55	0.70	1.82	0.65	0.64
	0	No change	11	13.53	6.60	20.46	1.15						
	**Perceived change of ADL**		***N***	**Mean**	**Lower** ***CI***	**Upper** ***CI***	**SRM**	**Cutoff**	**Sensitivity**	**Specificity**	**LR** **+**	**LR –**	**AUC**
Arm ROM	1	Mild improvement	13	21.20	11.22	31.19	1.25	17.16	0.62	0.73	2.26	0.53	0.67
	0	No change	12	11.58	4.53	18.63	0.97						
DT Gait speed	1	Mild improvement	13	0.14	0.08	0.19	1.38	0.13	0.69	0.73	2.54	0.42	0.67
	0	No change	12	0.10	0.03	0.16	0.86						

*ROM, range of motion; DT, dual-task; ADL, activities daily living; CI, confidence interval; SRM, standardized response mean; LR+, positive likelihood ratio; LR–, negative likelihood ratio; AUC, area under the curve*.

## Discussion

Our findings suggest that objective measures of Gait significantly improved with the ABC-C intervention in a group of 86 individuals with PD. In addition, we found small improvements in objective measures of APAs and Sway, as hypothesized. The effect size of objective measures was larger than the effect sizes of all clinical measures after the ABC-C intervention compared to the Education intervention. To our knowledge, this is the first study to systematically compare the responsiveness of objective measures on four different balance domains (Sway, APRs, APAs, and Gait) vs. clinical balance and gait measures to an exercise intervention.

Consistent with previous studies, including our original Agility Boot Camp training (10, 37), the current ABC-C intervention improved objective measures of gait, as well as of APAs. Gait pace (gait speed and foot strike angle), upper body movement during gait (arm ROM and trunk coronal ROM), and APA (peak ML acceleration and first-step ROM) measures showed significant improvements with the ABC-C intervention but not with the Education (active control) intervention. Interestingly, three of the four most discriminative measures to PD compared to age-matched control subjects in Gait (foot strike angle and arm ROM) and APAs (first-step ROM) improved with the ABC-C intervention (24). Thus, the ABC-C intervention seems to improve the most affected balance and gait signs in a group of people with moderate PD.

Of the four most sensitive objective mobility measures to PD, only turning did not improve with the ABC-C intervention. The lack of change in turning velocity may be related to the fact that the ABC-C intervention did not specifically focus on practicing turning, due to difficulty in maintaining safety with three to six subjects in the group exercise program. In addition, it is not clear if an increased velocity during turning would be a safe strategy in people with PD, as it has been shown that when turning faster, people with PD spend more time with the center of mass outside the base of support, a strategy that could be more prone to falls (38).

As hypothesized, postural responses to a perturbation did not improve after the ABC-C intervention. Previous exercise studies have reported improvements of postural responses (39–41), but these studies specifically trained postural responses to external perturbations. For example, previous studies used repetitive pulls to the participant's back (39) or repeated perturbation of a platform (40) or treadmill (41). Although the ABC-C intervention may have included postural perturbations induced by boxing with a contact of gloved fist onto a padded hand, these perturbations to both the boxer and the recipient of the punch (on glove) were relatively mild and could be anticipated by the participants. Studies showing improvements in postural stepping responses exposed subjects to many unexpected and stronger perturbations, and they used the same tests for training and assessing the effects of exercise (39–42).

Lastly, this study also provided MCID values for arm ROM during a single-task walk and gait speed during a dual-task walk, the only two measures significantly associated with perceived changes in Mobility or ADL. The MCID represents the smallest difference in score, which patients perceived as beneficial (9); thus, the value is very useful for assessing effects of a treatment. Both anchor-based approaches gave similar results, and the effect sizes for these two measures were large. Therefore, we considered a 21.2-degree change as the most appropriate MCID for arm ROM during a single-task walk and 0.14 m/s as the MCID for gait speed during a dual-task walk. Furthermore, 28 of 86 participants (32.6%) improved arm swing beyond the MCID of 21.2 degrees with the ABC-C intervention. In addition, the average change in improvement of gait speed in our PD cohort was close to 0.14 m/s MCID, and 44 of 86 participants (51.2%) improved beyond the MCID with the ABC-C intervention.

The clinical outcome measures were less sensitive to change with the ABC-C intervention compared to the objective measures (smaller effect sizes). In fact, we observed a small effect size only for all of the MDS-UPDRS (SRM_ABC−C_: total score = 0.25, Part II = 0.35, Part III = 0.20, and PIGD = 0.49), total score and APAs and Gait subscore of the Mini-BESTest (SRM_ABC−C_ = 0.29, 0.23, and 0.35, respectively), and the PDQ-39 total score and ADL subscore (SRM_ABC−C_ = −0.24 and −0.22), see Figure 3B. Our results are in keeping with previous studies investigating the effect of exercise in people with PD supporting that the change in objective measures was more sensitive to exercise intervention compared to clinical measures (10, 18, 19). Last, participants averaged 1.73 ± 7.72 points of changed improvement in the PDQ-39 ADL, lower than published MCID from 13.6 to 17.3 points for people with PD (43, 44). The lack of improvement in clinical or patient-reported outcomes may be related to the length of our study. In fact, participants are asked “how often have you had difficulty during the last month?” on the PDQ-39. A 6-week intervention period may be too brief to observe noticeable changes in clinical or perceived measures (8, 18, 45–49). In addition, as the ABC-C intervention was carried out as group exercise, including participants with different disease severity and cognitive abilities in the same group, the program may have been less challenging for people with milder disease severity. Thus, people with more severe symptoms or mildly impaired cognitive abilities may have benefited more from the ABC-C compared to people with PD with mild symptoms and intact cognition (20).

There are several limitations on this study that should be considered when interpreting the results. One limitation is that our larger cohort of people with PD used to identify the most discriminative measures of balance dysfunction included in this analysis is based on the baseline assessment of the participants included here (24). Another limitation was that we did not have a wash-out period; therefore, there could have been a carryover effect of exercise. However, although for few objective measures there was a trend toward a period effect, no objective measures actually showed a significant period effects (at *p* < 0.01). Lastly, only eight participants (9%) were assessed as Hoehn and Yahr stage IV, so results cannot be generalized to more severely affected people with PD. We did not collect fall data in our subjects or have a follow-up period to determine whether the effects of exercise lasted over time.

Further investigations with longer duration interventions, as well as a parallel design and a longer follow-up period, are needed to determine the longer-term effects of the ABC-C on balance and gait dysfunction. In addition, future interventions to improve balance in PD should also include training of multiple domains of balance, including APRs, standing balance on compliant surfaces and turning quality, as well as APAs and gait mobility. This study supports the use of objective measures of gait and balance, such as from wearable technology, by clinicians, as objective measures may be more sensitive to subtle improvements with exercise than clinical measures.

## Conclusion

This study showed that the ABC-C intervention improved only certain domains of balance control in people with PD even when these changes in objective measures were not reflected in clinical outcome measures. Specifically, gait pace (foot strike angle and gait speed), upper body movements during gait (arm and trunk ROM), and APAs (first-step length) were the most sensitive to change after the ABC-C intervention compared to the active control Education intervention. Among the clinical outcomes, patient-related outcomes, such as QOL, and balance also improved significantly but were not as sensitive to change as the objective measures. These findings suggest that clinicians should add objective measures of gait and balance, such as from wearable technology, before and after therapy interventions, as objective measures may be more sensitive to subtle changes than clinical rating scales.

## Data Availability Statement

The datasets generated for this study are available on request to the corresponding author.

## Ethics Statement

This study was carried out in accordance with the recommendations of the Oregon Health and Science University (OHSU) and Veterans Affairs Portland Health Care System (VAPORHCS) joint institutional review board (IRB) with written informed consent from all subjects. All subjects gave written informed consent in accordance with the Declaration of Helsinki. The protocol was approved by the OHSU (#4131) and the OHSU/VAPORHCS joint IRB (#8979).

## Author Contributions

NH: data analysis, drafting, and editing of the manuscript. VS: data analysis and editing of the manuscript. SJ: data analysis and editing of the manuscript. JL: statistical design, conceptualization of the study, and editing of the manuscript. PC-K and GH: study coordination, data collection, and editing of the manuscript. JN: conceptualization of the study and editing of the manuscript. NB: execution of the intervention and editing of the manuscript. LK: conceptualization of the study and methodology of the intervention and editing of the manuscript. FH: conceptualization of the study, obtained funding, and editing of the manuscript. MM: conceptualization of the study, supervising of the study, data collection, data analysis, and editing of the manuscript. All authors contributed to the article and approved the submitted version.

## Conflict of Interest

FH has an equity interest in APDM, a company that may have a commercial interest in the results of this study. This potential conflict of interest has been reviewed and managed by the Research and Development Committee at the VA Portland Medical Health Care System and Oregon Health and Science University. They have put in place a plan to help ensure that this research study is not affected by the financial interest. The remaining authors declare that the research was conducted in the absence of any commercial or financial relationships that could be construed as a potential conflict of interest.
